# Dystrophic Cutaneous Calcification and Metaplastic Bone Formation due to Long Term Bisphosphonate Use in Breast Cancer

**DOI:** 10.1155/2013/871917

**Published:** 2013-07-15

**Authors:** Ali Murat Tatlı, Seyda Gunduz, Sema Sezgin Göksu, Deniz Arslan, Mukremin Uysal, Cumhur İbrahim Başsorgun, Hasan Şenol Coşkun

**Affiliations:** ^1^Department of Medical Oncology, Faculty of Medicine, Akdeniz University, 07070 Antalya, Turkey; ^2^Department of Medical Oncology, Faculty of Medicine, Afyon Kocatepe University, 03200 Afyonkarahisar, Turkey; ^3^Department of Pathology, Faculty of Medicine, Akdeniz University, 07070 Antalya, Turkey

## Abstract

Bisphosphonates are widely used in the treatment of breast cancer with bone metastases. We report a case of a female with breast cancer presented with a rash around a previous mastectomy site and a discharge lesion on her right chest wall in August 2010. Biopsy of the lesion showed dystrophic calcification and metaplastic bone formation. The patient's history revealed a long term use of zoledronic acid for the treatment of breast cancer with bone metastasis. We stopped the treatment since we believed that the cutaneous dystrophic calcification could be associated with her long term bisphosphonate therapy. Adverse cutaneous events with bisphosphonates are very rare, and dystrophic calcification has not been reported previously. The dystrophic calcification and metaplastic bone formation in this patient are thought to be due to long term bisphosphonate usage.

## 1. Background

 The skeletal system is one of the most frequent areas of metastasis in breast cancer, and median survival is around 2 years in these patients [1]. Patients may suffer from serious skeletal problems due to bone metastases, and their quality of life may be severely impaired. Furthermore, these skeletal problems may render further treatment inapplicable and may have a detrimental effect on survival [2]. Zoledronic acid is a drug commonly used to prevent the complications of bone metastasis. However, there is no data on patients who have used zoledronic acid for longer than 5 years. There are safety data from phase 3 studies where more than 3000 patients with bone metastases used zoledronic acid for longer than 2 years. The most frequent adverse events due to zoledronic acid therapy are bone pain, nausea, fatigue, vomiting, pruritus, and a fever that may be caused by acute phase reactants due to infusion. However, there have been no adverse events reported on usage of a cumulative zoledronic acid dose for longer than 2 years [3]. Long term zoledronic acid therapy in patients with normal renal function, if no other nephrotoxic agents are used concomitantly, is highly safe with regard to the renal adverse events profile [4]. Osteonecrosis of the jaw is another adverse event due to zoledronic acid use; however, it is rarely seen. It is observed normally in just 1.3% of patients on long term therapy, whereas this adverse event may affect 61.8% of patients who have dental extractions or dental surgery [5]. In the present paper, we report a case of breast cancer with dystrophic calcifications on the skin after long term zoledronic acid usage. 

## 2. Case

A sixty-one-year old female with breast cancer presented with a rash around a previous operation site as well as a discharge lesion on the right chest wall in August 2010. The patient's history revealed a right modified mastectomy for breast cancer (T2N1M0) in 1996, followed by adjuvant chemotherapy, radiotherapy, and five years of tamoxifen treatment, consecutively. She was diagnosed with recurrent breast cancer with bone metastasis in 2003 via whole body scintigraphy and computerized tomography of the thorax. She was started on a treatment of zoledronic acid and letrozole. The patient used zoledronic acid for 5 years and clodronate for 2 years. 

**Figure 1:**
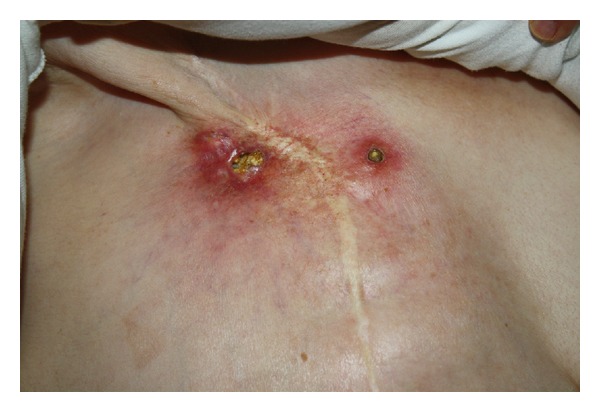


**Figure 2:**
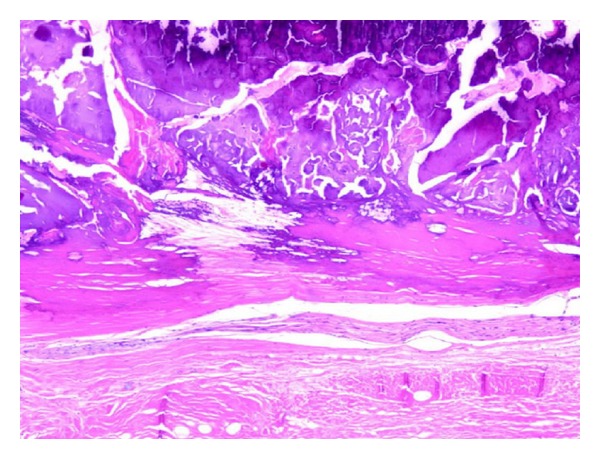
Fibroosseous lesion in dermis. There is a well-shaped, large fibroosseous metaplastic area (HEX50).

Biopsy of the lesion showed dystrophic calcification and metaplastic bone formation (Figure 1). Another biopsy was performed, and no malignant cells were observed on either of the two specimens (Figure 2). The lesion was excised by the plastic surgeon and reconstruction was performed. Pathology reported granulation tissue with acute inflammatory attack, fibrosis, and dystrophic calcification. There was no recurrence of the tumor in the surgical material.

 The dystrophic calcifications and metaplastic bone formation on the patient's front chest wall were evaluated and determined to be associated with bisphosphonate therapy. Another whole body scintigraphy was performed and revealed increased activity in multiple bone foci. No metastases were observed on the thorax or abdominal CTs. We decided to stop bisphosphonate therapy and continue the patient on letrozole. 

## 3. Discussion

Calcinosis cutis is a condition in which insoluble calcium salt deposits accumulate on the skin and subcutaneous tissue. This syndrome consists of 5 different groups as dystrophic calcification, metastatic calcification, iatrogenic calcification, idiopathic calcification, and calciphylaxis. Dystrophic calcification occurs as the result of local tissue injury and/or a persisting local abnormality, such as a wound. In the context of a wound, a continued inflammatory state, the presence of nonviable or necrotic tissue, or alterations in the cellular or extracellular components (collagen, elastin, proteoglycans, etc.) may precipitate calcification. In dystrophic calcification, calcium and phosphate metabolism and their serum levels are normal, while the internal organs remain unaffected. In metastatic calcification, the calcium and/or phosphate metabolism is abnormal, and hypercalcaemia or hyperphosphatemia can be seen. This may lead to the calcification of normal tissues. Iatrogenic calcinosis cutis may occur as a result of intravenous calcium gluconate treatment. Calciphylaxis occurs due to the accumulation of small calcium deposits on the dermis and subcutaneous fatty tissue vessel walls [6, 7]. The majority of dystrophic calcifications reported in the literature are cases of ulcerous lesions with accompanying chronic venous failure of the lower extremities. Other conditions where dystrophic calcifications are observed are in connective tissue diseases, traumas, panniculitis, porphyria cutanea tarda, pseudoxanthoma elasticum, cutaneous malignancies, and in Ehler-Danlos syndrome [8, 9]. There are not any previous reports in the literature of dystrophic calcification cases that are thought to be associated with the long term therapy of zoledronic acid. There is only a single case report of breast cancer, by Riahi and cohen, where multiple subcutaneous osteoma formations were observed on the face after a year of alendronate usage for treatment of osteoporosis [10]. In our patient, dystrophic calcification and metaplastic bone formation occurred on the mastectomy scar. Calcium and phosphate levels were normal. We believed that dystrophic calcification was associated with the long term therapy of zoledronic acid for bone metastasis. Tissue healing was achieved by local excision followed by reconstructive surgery by plastic surgeons. Even though localized treatment is suggested for dystrophic calcification, definitive treatment recommendations are not clear. It was reported in an animal study by Grases et al. that myoinositol-hexaphosphate-based creams may prevent the formation and progress of dystrophic calcification [11]. The patient is still alive with no recurrence of either breast cancer or dystrophic calcification.

A recent study comparing two different regimes of zoledronic acid usage showed that using zoledronic acid quarterly (4 mg q 12 weeks) was not inferior to monthly usage (4 mg q 4 weeks) after a year of prior zoledronic acid treatment [12]. The alternative low dosage regimen may be a choice to prevent these adverse effects. 

 Zoledronic acid, which is an established therapy for reducing the risk of skeletal related events in patients with bone metastasis, may be related to cutaneous dystrophic calcification and metaplastic bone formation with long term usage.
